# Functional glutamate transporters are expressed in the carotid chemoreceptor

**DOI:** 10.1186/s12931-020-01468-z

**Published:** 2020-08-08

**Authors:** Chaohong Li, Lu Huang, Xianglei Jia, Baosheng Zhao, Lingyun Chen, Yuzhen Liu

**Affiliations:** 1 The First Affiliated Hospital of Xinxiang Medical University, Henan Key Laboratory of Neural Regeneration and Repairment, Henan Neurology Institute, 88 Jiankang Road, Weihui, 453100 Henan China; 2 Department of Thoracic Surgery, The First Affiliated Hospital of Xinxiang Medical University, Weihui, Henan China; 3 Operating Room, The First Affiliated Hospital of Xinxiang Medical University, Weihui, Henan China

**Keywords:** Carotid body, Glutamate, Vesicular glutamate transporter, Excitatory amino acid transporter, Cyclic intermittent hypoxia

## Abstract

**Background:**

The carotid body (CB) plays a critical role in cyclic intermittent hypoxia (CIH)-induced chemosensitivity; however, the underlying mechanism remains uncertain. We have demonstrated the presence of multiple inotropic glutamate receptors (iGluRs) in CB, and that CIH exposure alters the level of some iGluRs in CB. This result implicates glutamatergic signaling in the CB response to hypoxia. The glutamatergic neurotransmission is not only dependent on glutamate and glutamate receptors, but is also dependent on glutamate transporters, including vesicular glutamate transporters (VGluTs) and excitatory amino acid transporters (EAATs). Here, we have further assessed the expression and distribution of VGluTs and EAATs in human and rat CB and the effect of CIH exposure on glutamate transporters expression.

**Methods:**

The mRNA of VGluTs and EAATs in the human CB were detected by RT-PCR. The protein expression of VGluTs and EAATs in the human and rat CB were detected by Western blot. The distribution of VGluT3, EAAT2 and EAAT3 were observed by immunohistochemistry staining and immunofluorescence staining. Male Sprague-Dawley (SD) rats were exposed to CIH (FIO_2_ 10–21%, 3 min/3 min for 8 h per day) for 2 weeks. The unpaired Student's *t*-test was performed.

**Results:**

Here, we report on the presence of mRNAs for VGluT1–3 and EAAT1–3 in human CB, which is consistent with our previous results in rat CB. The proteins of VGluT1 and 3, EAAT2 and 3, but not VGluT2 and EAAT1, were detected with diverse levels in human and rat CB. Immunostaining showed that VGluT3, the major type of VGluTs in CB, was co-localized with tyrosine hydroxylase (TH) in type I cells. EAAT2 and EAAT3 were distributed not only in type I cells, but also in glial fibrillary acidic protein (GFAP) positive type II cells. Moreover, we found that exposure of SD rats to CIH enhanced the protein level of EAAT3 as well as TH, but attenuated the levels of VGluT3 and EAAT2 in CB.

**Conclusions:**

Our study suggests that glutamate transporters are expressed in the CB, and that glutamate transporters may contribute to glutamatergic signaling-dependent carotid chemoreflex to CIH.

## Background

Patients with obstructive sleep apnea (OSA) experience repetitive nocturnal upper airway obstructions, each accompanied by oscillations in oxygen saturation, causing cyclic intermittent hypoxia (CIH). One consequence of OSA is daytime hypertension, as increased arterial pressure persists after oxygen saturation has returned to normal. Three epidemiologic investigations have established a link between OSA and hypertension [1–3], but have not revealed the mechanisms by which nocturnal upper airway obstruction leads to elevated arterial pressure. Animal models have, however, linked CIH, such as is experienced during sleep by OSA patients, to altered hemodynamics during normoxia. Fletcher et al. [4] exposed rats to intermittent hypoxia for 7 h each day for 5 weeks, resulting in significant increase in arterial pressure. Notably, in this model the sympathetic nervous system was necessary for increased pressure, as ablation of the renal nerve prior to the exposure to CIH prevented the rise in arterial pressure. Further study showed that denervation of the peripheral chemoreceptor carotid body (CB) prevented increased arterial pressure after CIH exposure [5, 6].

The CB is a small cluster of peripheral chemoreceptor located bilaterally near the bifurcation of the common carotid artery, and is made up of two major cells: the glomus cells (type I cells), and sustentacular cells (type II cells). Glomus cells derived from the neural crest are mainly oxygen sensitive cells, and contain a variety of neurotransmitters and neuromodulators. These neurotransmitters and neuromodulators include acetylcholine [7], ATP [8], dopamine [9, 10], endothelin-1 [11] and angiotensin II [12, 13]. The carotid chemoreceptor mediates the body’s integrated responses to hypoxia. Reductions in inspired oxygen are translated nearly instantly into afferent nerve signals. These signals are trafficked to the nucleus tractus solitarius, and then relayed to other centers in the brain, resulting in increased respiration and a coordinated cardiovascular response that attempts to preserve oxygen delivery. This response requires the oxygen sensitive cells (type I cells) in carotid chemoreceptor to detect the changes of oxygen levels, resulting in afferent activation of the carotid sinus nerve. Repetitive hypoxia results in plasticity of the chemoreceptor response [14, 15], so that afferent nerve traffic is increased under intermittent hypoxia environment. Considerable evidences suggest that exposure to CIH has lasting effects on the CB, inducing a specific form of carotid chemoreflex plasticity termed hypoxic acclimatization [14, 16]. Among the mechanisms that have been proposed to underlie chemoreflex plasticity are: alterations in the properties of the oxygen-sensitive K^+^ channels [17]; changes in expression of redox-sensitive proteins in glomus cells [18]; altered expression in the CB of neuromodulators such as endothelin I [19] or angiotensin II [13, 20]; and altered expression in CB of neurotransmitters, such as dopamine [21, 22]. More recently, we have found that multiple ionotropic glutamate receptors exist in the rat CB, and that exposing Sprague-Dawley (SD) rats to CIH for 8 h/day for 3 weeks significantly enhanced the expression level of N-methyl-D-aspartate Receptor 1 (NMDAR1) and alpha-amino-3-hydroxy-5-methyl-4-isoxazole propionic acid receptor 1 (AMPAR1) [23, 24], two subtypes of inotropic glutamate receptor in rat CB. These findings suggest glutamatergic transmission from glomus cells may underlie some forms of chemoreflex plasticity in response to CIH.

Glutamate is a major excitatory neurotransmitter in the brain and is specifically accumulated into synaptic vesicles by a family of proteins known as vesicular glutamate transporters (VGluTs) [25]. Thus, VGluTs are so far the most specific biomarkers for neurons and other cells using glutamate as a neurotransmitter. Following presynaptic depolarization, which results in cytosolic Ca^2+^ increases, glutamate is released from VGluT-containing synaptic vesicles by exocytotic membrane fusion into the synaptic cleft. Upon release in the synaptic cleft, glutamate plays an essential role in glutamatergic signaling transmission by binding to its receptors on the postsynaptic membrane. To keep synaptic concentration of glutamate below excitotoxic level and to maintain temporal specificity of glutamate mediated signaling, glutamate excitatory amino acid transporters (EAATs) take up glutamate from the synaptic cleft into adjacent glial cells or pre-synaptic cells [26]. There are three subtypes of VGluTs: VGluT1, VGluT2, VGluT3. Additionally there are five subtypes of EAATs: EAAT1, EAAT2, EAAT3, EAAT4 and EAAT5. Evidence suggests that either VGluTs or EAATs are involved in the regulation of glutamatergic transduction by controlling glutamate dynamic levels in synaptic cleft [27].

We previously demonstrated that multiple mRNAs of VGluTs and EAATs were expressed in the rat CB, indicating that glutamate might be stored as a neurotransmitter, then released and taken up in the CB. Here, we have further assessed the mRNA expression of VGluTs and EAATs in human CB and the protein expression of VGluTs and EAATs in the rat and human CB. Moreover, we have investigated the distribution of VGluTs and EAATs in the rat CB and the effect of CIH exposure on the expression level of glutamate transporters.

## Methods

### Reagents and antibodies

RNAlater and HotStarTaq® Master Mix Kit were purchased from Qiagen (Valencia, CA, USA). TRizol Reagent was purchased from Thermo Fisher Scientific (Waltham, MA, USA). 5 X All-In-One RT MasterMix (with AccuRT Genomic DNA Removal Kit) was purchased from Applied Biological Materials Inc. (Richmond, BC, Canada). RIPA buffer, BCA Protein Assay kit, Chemiluminescent Substrate Detection kit and HRP-linked goat anti mouse IgG were purchased from Boster (Wuhan, China). Protease inhibitor cocktail and phosphatase inhibitors were purchased from Roche (Basel City, Switzerland). Rabbit antibodies against VGluT1, EAAT2, tyrosine hydroxylase (TH), glial fibrillary acidic protein (GFAP), and mouse antibody against VGluT3 were all purchased from Abcam (Cambridge, UK). Rabbit antibodies against VGluT2, EAAT1, EAAT3, β-actin and mouse antibody against GFAP, Alexa Fluor 488 goat anti-rabbit IgG and Alexa Fluor 555 goat anti-mouse IgG were purchased from Cell Signaling Technology (Danvers, MA, USA). Diaminobenzidine, isoflurane and mouse anti-TH antibody were ordered from Sigma (St Louis, MO, USA). Polink-2 plus® Polymer HRP Detection System was obtained from ZSGB-BIO (Beijing, China).

### Animals and cyclic intermittent hypoxia exposure

Male SD rats were obtained from Beijing Vital River Laboratory Animal Technology Co, Ltd. (Beijing, China), aged 8 weeks and weighed 240–250 g at entry into the protocol. Rats were housed under room temperature and standard humidity (50 ± 5%) with a 12 h day/night circle with laboratory chow and water ad libitum. All procedures performed in this study were in accordance with national animal research regulations, and all animal experimental protocols were approved by the Institutional Animal Ethics Committee at the First Afflicted Hospital of Xinxiang Medical University.

CIH exposure and control procedures were similar with previously report procedures [23]. In brief, rats in their home cages were placed inside the Oxycycler Model A84XOV (Biospherix, Redfield, NY, USA) hypoxia system and exposed to CIH or room air. O_2_ fraction (FIO_2_) in each chamber was monitored and regulated by two timer-controlled valves. The chamber was flushed with 100% N_2_ to inspired FIO_2_ nadir of 10% for 3 min. The FIO_2_ gradually returned to 21% over the remainder of each cycle. The exposure cyclic was repeated every 6 min for 8 h/day from 8:30 to 16:30 for 14 consecutive days during rat sleeping hours. For the control group, the control rats underwent the same exposure, but the chamber was flushed with compressed air. After completion of CIH exposure, all animals were assigned to the following studies.

### Human carotid body

Human surgical specimens were obtained from The First Affiliated Hospital of Xinxiang Medical University with consent from patients (granting approval number: 2016008). Human CB specimen was obtained from a patient with left CB paraganglioma and human cerebral cortex tissue was obtained from a patient with craniocerebral trauma. The protocol related to human subjects was conducted in accordance with the declaration of Helsinki, and approved by the Ethics Committee of The First Affiliated Hospital of Xinxiang Medical University.

### Rat carotid body harvest

After anesthesia was achieved by inhalation of 2% isoflurane, the rat was decapitated and the carotid bifurcations were rapidly removed and placed in 95% O_2_–5% CO_2_ statured ice-cold PBS. The CBs were dissected and then immediately soaked in RNAlater and stored at − 80 °C until analyzed. The approximate time from euthanasia to removal of the CB to placement in RNAlater was about 4 min.

### RNA extraction and RT-PCR

Total RNA was extracted from human CB specimen using Trizol-reagent. For the reverse transcription (RT), 500 ng of total RNA reversely transcribed into cDNA after gDNA removal using 5 X All-In-One RT MasterMix (with AccuRT Genomic DNA Removal Kit), in accordance to the manufacturer’s protocol. Instead of RT MasterMix, DEPC-H_2_O was used in reverse transcription reaction to obtain a negative cDNA control. The mRNA expression level was detected by PCR in a Veriti® 96-well Thermal cycler (Applied Biosystems, Foster City, CA, USA) using HotStarTaq® Master Mix Kit. According to the manufacturer’s protocol, 10 μl of HotStarTaq Master Mix, 1 μl of gene-specific primer pairs and 2 μl of cDNA were mixed together to produce a final volume of 20 μl. The PCR reactive conditions were 95 °C for 10 min, followed by 40 cycles at 94 °C for 50 s, then annealing temperature for 50 s and 72 °C for 1 min, then ending at 72 °C for 10 min. An equal volume of the negative cDNA control was used in the PCR as negative PCR control. The PCR product was loaded into 1.2% agarose gel. A total of 3 technical replicates were conducted. All primers were exon spanning and designed using Primer-BLAST (https://www.ncbi.nlm.nih.gov/). Details of all primers are listed in Table 1.

**Table 1:** Sequence of human primers used in RT-PCR experiment. VGluT: vesicular glutamate transporter, EAAT: excitatory amino acid transporter, Tm: temperature, F: forward primer, R: reverse primer.

Gene	Accession	Primer	PCR Cycle no.	Annealing Tm (^0^C)
VGluT1	**NM_020309.4**	F: 5’-TGCCTCTCAGGGGTAGTGAA-3’	45	53.5
		R: 5’-ATCAATCCCTGGAATGGCGG-3’		
VGluT2	**NM_020346.2**	F: 5’-GGGAGACAATCGAGCTGACG-3’	45	56.5
		R: 5’-TGCAGCGGATACCGAAGGA-3’		
VGluT3	**NM_139319.3**	F: 5’-CTCAGAGGTGCCCCTCATTC-3’	45	53.5
		R: 5’-TGCATAAACCGGCAAAGATGTG-3’		
EAAT1	**NM_004172.5**	F: 5’- AGCAGGGAGTCCGTAAACG-3’	35	51.8
		R: 5’-AGCATTCCGAAACAGGTAACTTT-3’		
EAAT2	**NM_004171.4**	F: 5’-CCTGACGGTGTTTGGTGTCAT-3’	35	54.4
		R: 5’-CAAGCGGCCACTAGCCTTAG-3’		
EAAT3	**NM_004170.5**	F: 5’-TTTCTGTACCACTCTCATTGCTG-3’	35	51.8
		R: 5’-TCCACCGTACTGACTTCAGGG-3’		
GAPDH	**NM_002046.7**	F: 5’-GCAGGGGGGAGCCAAAAGGGT-3’	40	53
		R: 5’-TGGGTGGCAGTGATGGCATGG-3’		

### Protein extraction and western blot analysis

Protein was extracted from 16 CBs pooled from eight Control or eight CIH rats and human CB specimen. Tissues were homogenized with RIPA buffer containing phosphatase inhibitors and protease inhibitor cocktail. The supernatant was collected after centrifugation at 12,000 g/min for 10 min at 4 °C and protein concentration was detected by BCA protein assay kit. 35 μg of protein was loaded into 8% SDS-PAGE gel, and then transferred to PVDF membrane (Millipore, Darmstadt, HE, Germany) at 200 mA for 2 h. After blocking with 5% skim milk, the membrane was probed with primary antibodies including: rabbit anti-VGluT1 (1:1000), anti-VGluT2 (1:1000), anti-EAAT1 (1:1000), anti-EAAT2 (1:1000), anti-EAAT3 (1:1000), β-actin (1:1000) and mouse anti-VGluT3 (1:1000) antibodies. The membrane was then washed with TBS-T and incubated with second HRP-linked goat anti rabbit IgG (1:8000) or HRP-linked goat anti mouse IgG (1:5000) antibodies at room temperature for 1 h. After incubation, the membranes were washed three times with TBS-T and immersed in Chemiluminescent Substrate Detection kit and detected by AmershamTM Imager 600 system (GE Healthcare Bio-Sciences, Pittsburgh, PA, USA), in accordance to the manufacturer’s instruction. A total of 3 technical replicates were conducted.

### Immunohistochemistry staining

Rats were fixed with 4% neutral buffered formalin and the CBs were removed. The paraffin-embedded CB sections prepared by Shandon™ Finesse™ 325 Microtomes (Thermo Fisher Scientific, Waltham, USA) at 3 μm thickness were used. The sections were deparaffinized by being heated at 65 °C for 1 h and cleared through xylene solution, then rehydrated through washes in decreasing grades of ethanol (100, 95, 80, and 60%) for 3 min each. After washing with PBS, the slides were incubated in citrate buffer (pH 6.0) for 15 min at 100 °C to retrieve antigen. Then, sections were immersed to 3% H_2_O_2_ to block endogenous peroxidase activity. After being blocked with 10% goat serum, sections were incubated overnight at 4 °C with following primary antibodies including: rabbit anti-EAAT2 (1:200), rabbit anti-EAAT3 (1:50) and mouse anti-VGluT3 (1:100). After washing three times in PBS containing 0.2% Triton X-100, sections were incubated with Polink-2 plus® Polymer HRP Detection System, in accordance with the manufacturer’s instruction. Finally, sections were washed, and the antibody-antigen complex was visualized by incubating the sections with 0.01% H_2_O_2_ and 0.05% diaminobenzidine to yield a reddish-brown crystalline product. Negative staining control was prepared by omitting the primary antibody. The staining was examined through the Nikon H600L microscope and photographed with Nikon digital camera DS-Fi1c (Nikon, Tokyo, Japan).

### Double immunofluorescence staining

CB paraffin sections prepared as above were exposed to a mixture of two primary antibodies as follows: rabbit anti-EAAT2 (1:50) with mouse anti-GFAP (1:200); rabbit anti-EAAT2 (1:50) with mouse anti-TH (1:2000); rabbit anti-EAAT3 (1:50) with mouse anti-TH (1:2000); rabbit anti-EAAT3 (1:50) with mouse anti-GFAP (1:200); mouse anti-VGluT3 (1:100) with rabbit anti-TH (1:100); mouse anti-VGluT3 (1:100) with rabbit anti-GFAP (1:100). After washing, the slides were incubated with a mixture of Alexa Fluro 488 goat anti-rabbit IgG (1:400) and Alexa Fluro 555 goat anti-mouse IgG (1:400). Negative staining control was prepared by omitting the primary antibody. The staining was examined with the Axio Observer A1 microscope and photographed with the AxioCam MRc5 camera (Carl Zeiss, Gottingen, Germany).

### Statistical analysis

The data were presented as means ± S.D. Statistical evaluation was conducted by unpaired Student’s *t*-test. *P* < 0.05 was considered significant.

## Results

### mRNAs of glutamate transporters in human CB and protein expression of glutamate transporters in the CB

**Fig. 1:**
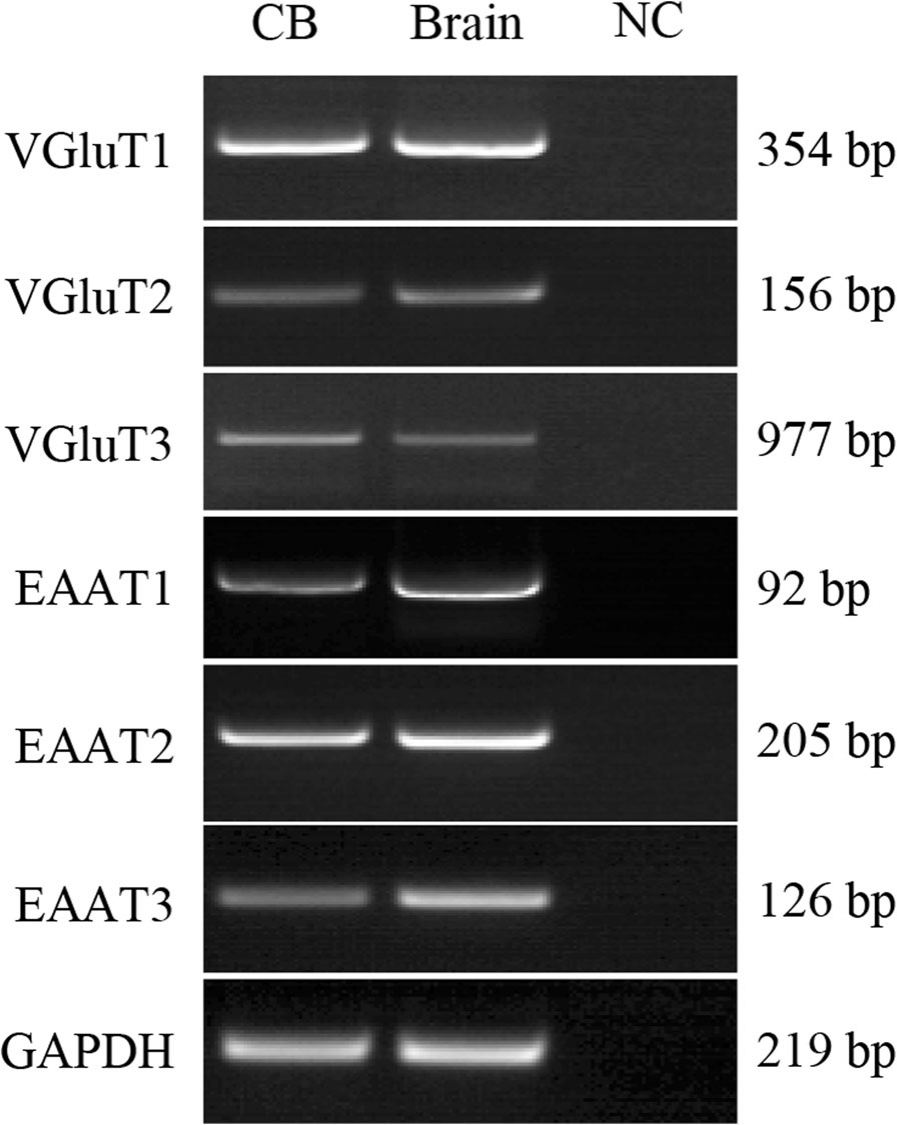
Expression of VGluTs and EAATs mRNAs in human CB. RT-PCR showed expression of mRNAs of VGluTs and EAATs in human CB (CB, left lane). RNA extracted from human brain cerebral cortex was used as positive control (Brain, middle lane). The DEPC-H_2_O, instead of RNA, was used in RT reaction to obtain a negative cDNA control. An equal volume of the negative cDNA control was used in the PCR as PCR negative control (NC, right lane). VGluT: vesicular glutamate transporter; EAAT: excitatory amino acid transporter

**Fig. 2:**
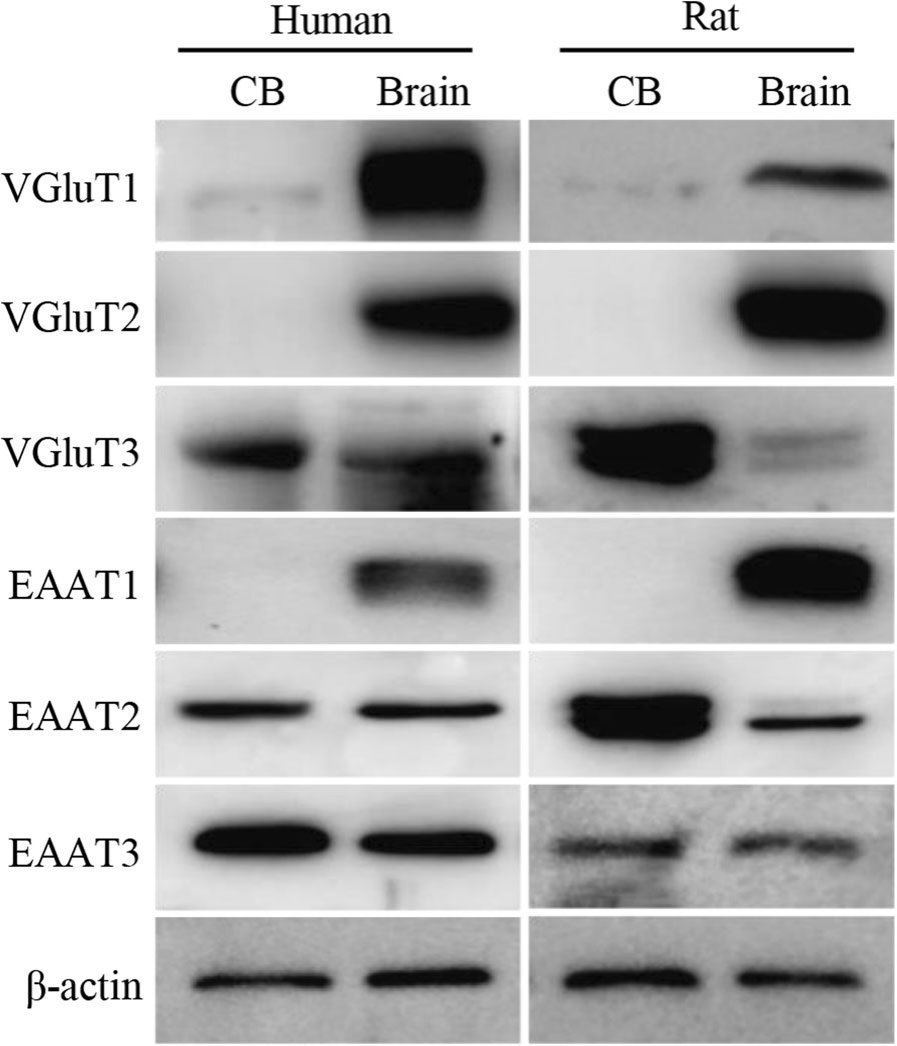
Expression of VGluTs and EAATs proteins in human and rat CB. Western blot showed expression of proteins of VGluTs and of EAATs in rat and human CB (CB, left lane). Protein extracted from human and rat brain cerebral cortex was used as positive control (Brain, right lane). VGluT: vesicular glutamate transporter; EAAT: excitatory amino acid transporter

We previously found that mRNAs of VGluT1–3 and EAAT1-3 were expressed in the rat CB. Here, we have extended the study to further determine whether these glutamate transporter mRNAs are expressed in human CB. RT-PCR was performed and the results are shown in Fig. 1. All PCR-amplified transcripts of VGluT1–3 and EAAT1–3 were detected in human CB as well as brain cerebral cortex, the latter was used as positive control, indicating that human CB expresses mRNAs of VGluT1–3 and EAAT1–3. These results are consistent with our previous findings in the rat CB. In an effort to further identify whether these glutamate transporter mRNAs are translated into the proteins accordingly in the CB, we conducted the western blot to examine the protein expression of these glutamate transporters in rat and human CB tissue lysates. As shown in Fig. 2, the proteins of VGluT1–3 and EAAT1–3, which were all detected in both human and rat brain samples, served as positive controls; however, not all of these protein bands were visible in human and rat CB. VGluT1, VGluT3, EAAT2 and EAAT3, rather than VGluT2, EAAT1 were detected at different levels in human and rat CB. Compared to individual brain control, the expression levels of VGluT3, EAAT2 and EAAT3 were relatively high in human and rat CB, whereas VGluT1 was expressed at a much lower level in human and rat CB.

### Distribution of VGluT3, EAAT2 and EAAT3 in the rat CB

**Fig. 3:**
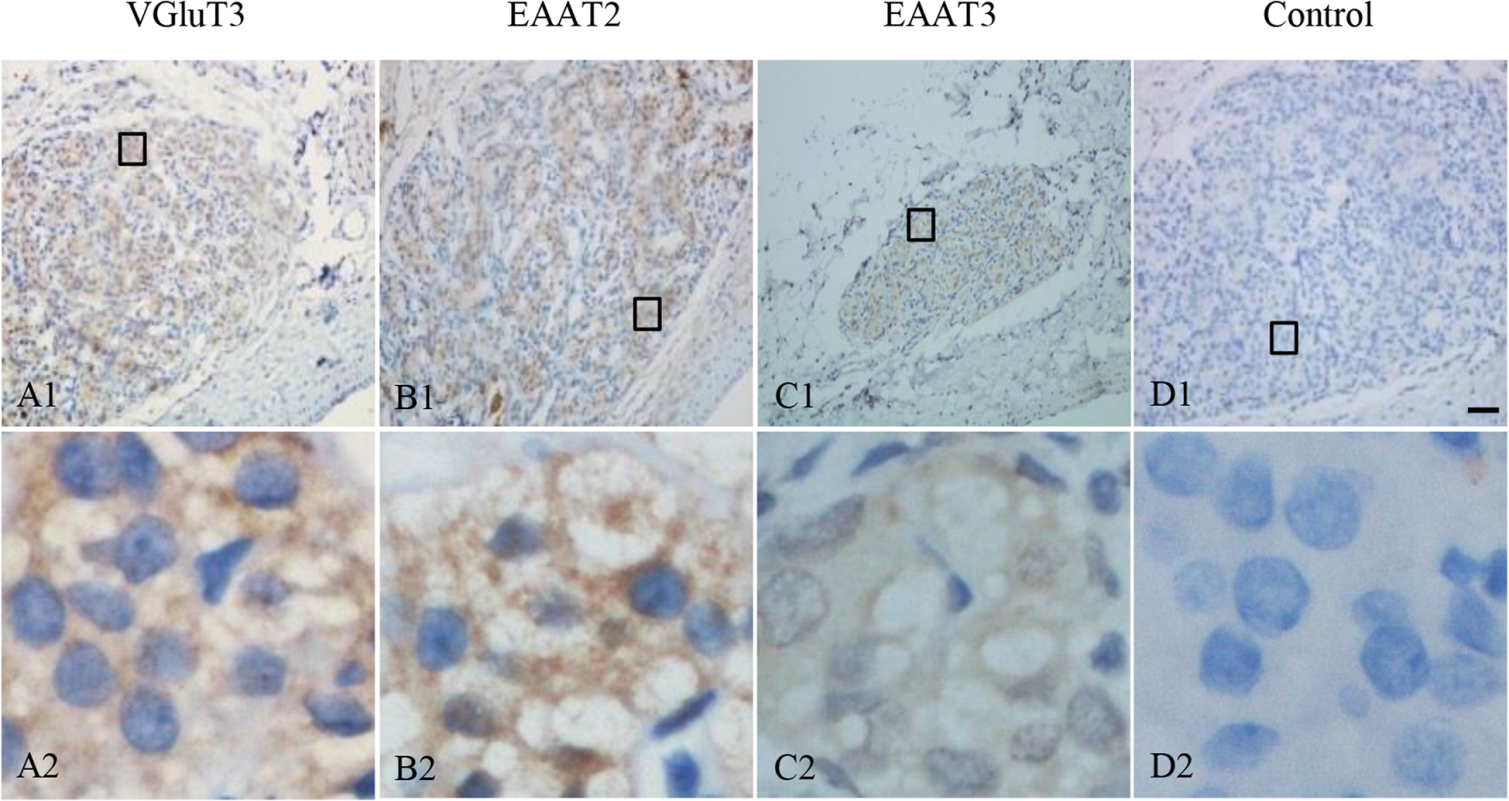
Immunohistochemical staining of VGluT3, EAAT2 and EAAT3 in the rat CB. Immunohistochemical staining showing the location of VGluT3 (A1–2), EAAT2 (B1–2) and EAAT3 (C1–2) in rat CB. D1-D2 are negative staining control obtained by omitting the primary antibody. A2-D2 are higher magnifications images of rectangle areas in A1-D1, respectively. The brown staining represents the glutamate transporter, while the blue staining represents hematoxylin-stained cell nuclei. VGluT: vesicular glutamate transporter; EAAT: excitatory amino acid transporter. Scale Bar = 50 μm for all images

To elucidate the distribution of VGluT3, EAAT2 and EAAT3, which were all detected by western blots, in the CB, immunohistochemistry was performed. Representative photomicrographs in Fig. 3 showed positive immunostaining of VGluT3, EAAT2 and EAAT3 in rat CB. The immunoreactivity of VGluT3 (Fig. 3A1-A2), EAAT2 (Fig. 3B1-B2) and EAAT3 (Fig. 3C1-C2) was ubiquitously distributed in the clustering glomeruli in rat CB. The representative negative control in which the primary antibody was replaced by PBS containing 3% normal goat serum did not yield specific staining in rat CB (Fig. 3D1-D2).

### Cellular localization of VGLUT3, EAAT2 and EAAT3 in the rat CB

**Fig. 4:**
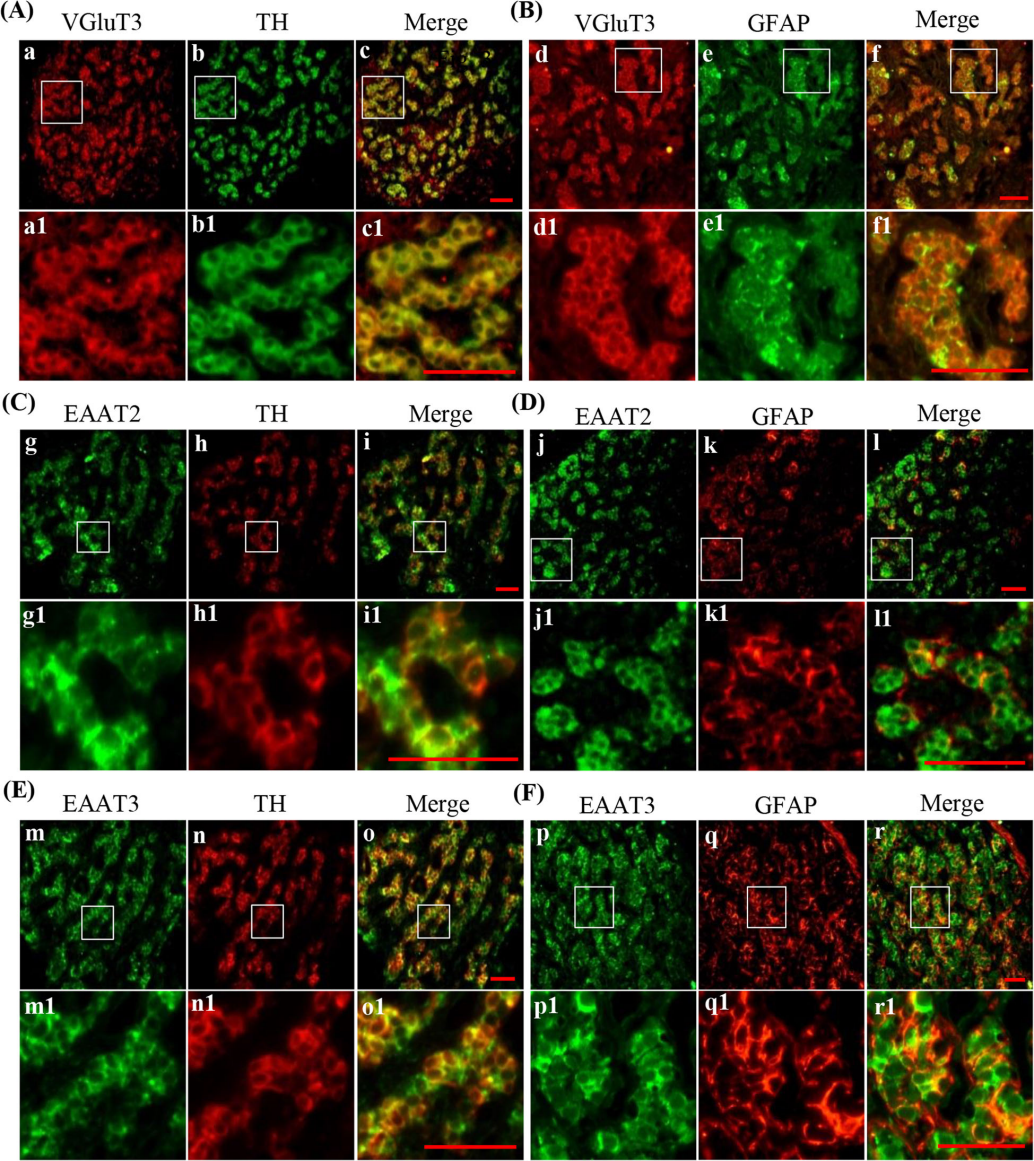
Cellular distribution of VGluT3, EAAT2 and EAAT3 in the rat CB. **A** Double immunofluorescence staining of VGluT3 (red) and tyrosine hydroxylase (TH, green) in rat CB (a-c1). **B** Double immunofluorescence staining of VGluT3 (red) and glial fibrillary acidic protein (GFAP, green) in rat CB (d-f1). **C** Double immunofluorescence staining of EAAT2 (green) and TH (red) in rat CB (g-i1). **D** Double immunofluorescence staining of EAAT2 (green) and GFAP (red) in rat CB (j-l1). **E** Double immunofluorescence staining of EAAT3 (green) and TH (red) in rat CB (m-o1). **F** Double immunofluorescence staining of EAAT3 (green) and GFAP (red) in rat CB (p-r1). a1-r1 are higher magnifications images of rectangle areas in a-c, respectively. TH: the CB type I cells marker; GFAP: the CB type II cells marker; VGluT: vesicular glutamate transporter; EAAT: excitatory amino acid transporter. Scale bar = 25 μm for all images

To further ascertain the cellular localization of VGluT3, EAAT2 and EAAT3 in the CB, double immunofluorescence was performed. TH was used as a glomus cell marker and GFAP was used as a type II cell marker. Double immunofluorescence of VGluT3 with TH in consecutive sections, as shown in Fig. 4A, revealed that VGluT3 immunoreactive signal was clearly observed in TH-positive glomus type I cells. Double staining of VGluT3 and GFAP (Fig. 4B) showed that VGluT3 immunoreactive signal was not obviously detectable in GFAP-positive type II cells, indicating that VGluT3 may be mainly distributed in type I cells of rat CB. Double staining of EAAT2 with TH (Fig. 4C) or GFAP (Fig. 4D) showed that some immunoreactive signals of EAAT2 were detected in TH positive type I cells and in GFAP positive type II cells, indicating that EAAT2 distributes both Type I and Type II cells in rat CB. Double staining of EAAT3 with TH (Fig. 4E) or GFAP (Fig. 4F) showed that immunoreactive signals of EAAT3 were merged with TH and GFAP, suggesting that EAAT3 is distributed both in type I and type II cells in rat CB.

### Effect of CIH exposure on VGLUT3, EAAT2 and EAAT3 expression in the rat CB

**Fig. 5:**
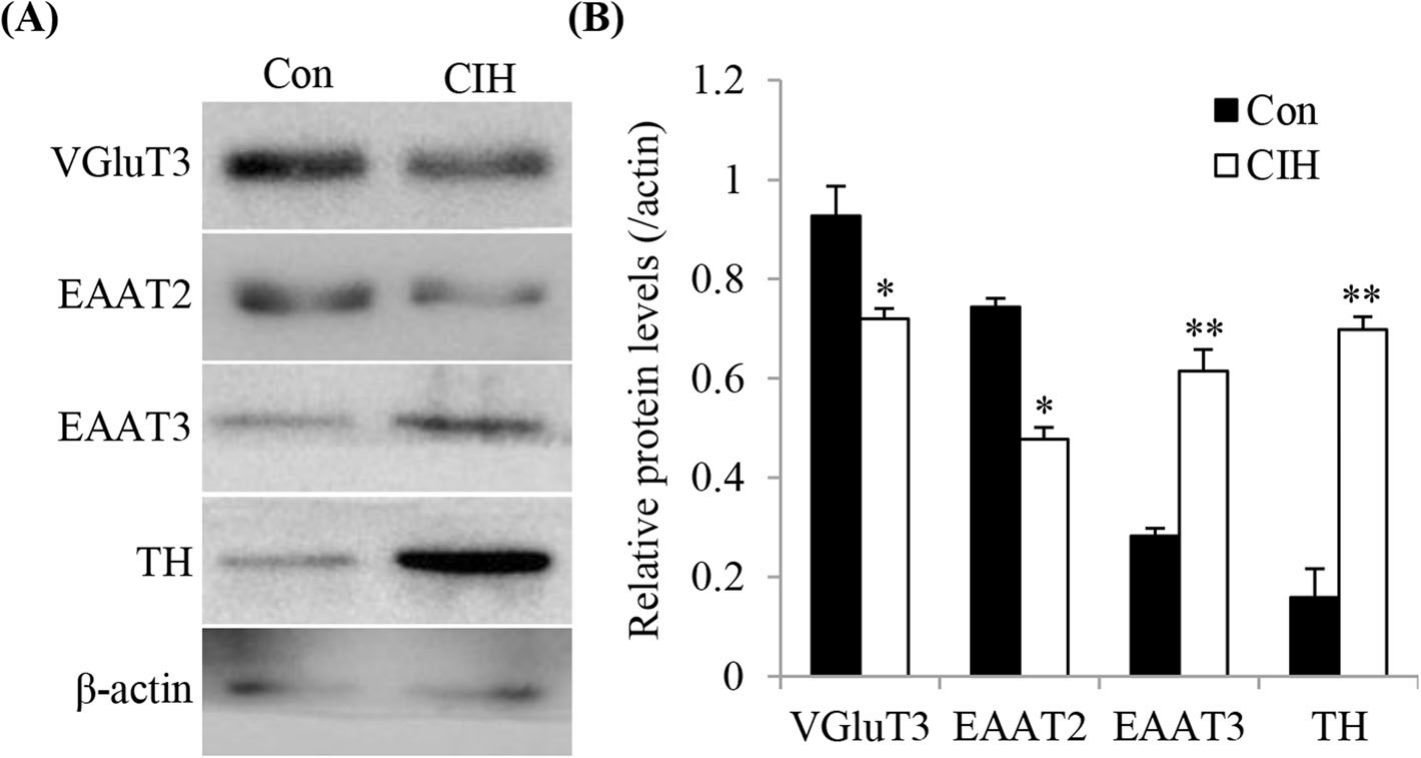
CIH treatment increases the EAAT3 protein level and decreases the VGluT3 and EAAT2 protein level in the rat CB. **a** Western blot showed protein expression level of VGluT3, EAAT2 and EAAT3 in the rat CB of Con and CIH group rats. **b** Statistical analysis of VGluT3, EAAT2 and EAAT3 protein level in the rat CB after CIH treatment. The data were presented as means ± S.D. The standard deviation bars represent the standard deviation from 3 technical replicates. *n* = 8 in each group, *P < 0.05, **P < 0.01

Glutamatergic signaling is involved in chemoreflex plasticity of CB in reaction to CIH [23, 24]. Therefore, to further examine the effect of CIH exposure on VGluT3, EAAT2 and EAAT3 protein expression levels, western blots were performed with the lysates of the CBs harvested from Control (Con) and CIH-exposed rats. As shown in Fig. 5, CIH reduced protein expression levels of VGluT3 about 0.27 fold (CIH vs Con, *P* < 0.05) and EAAT2 about 0.36 fold (CIH vs Con, *P* < 0.05), when compared with the control level. EAAT3 was augmented about 1.07 fold (CIH vs Con, *P* < 0.01) after CIH exposure, when compared with the control level. TH was used a positive control to show the CB response to hypoxia. These data indicate that CB chemoreflex plasticity to hypoxia might be modulated by alteration of glutamate transporter level.

## Discussion

We previously found that rat CB expresses mRNAs of VGluT1–3 and EAAT1–3 [24]. In this study, we demonstrated that mRNAs of all these glutamate transporters are also expressed in human CB. We further found that among the glutamate transporters, the proteins of VGluT1 and 3, EAAT2 and 3, rather than VGluT1 and EAAT1 were detectable with diverse levels in human and rat CB. The expression of VGluT3, EAAT2 and 3 was enriched in human and rat CB. In contrast, VGluT1 was expressed at quite a low level in the CB when compared with the brain. Moreover, we determined that VGluT3 is mainly localized in type I cells, while EAAT2 and EAAT3 are distributed in type I cells and type II cells of the CB. Furthermore, we found that CIH exposure elevated the protein level of EAAT3 as well as TH, but attenuated the levels of VGluT3 and EAAT2 in the CB. Taken together, these results coupled with our previous studies indicate that glutamate secretion and uptake systems may occur in the CB, and glutamate transporters may contribute to glutamatergic signaling-dependent carotid chemoreflex to CIH.

The CB is an important peripheral chemoreceptor that senses the changes of oxygen, carbon dioxide and pH levels in the arterial blood. It is reported that intermittent hypoxia leads to CB chemoreceptor plasticity [14], however, the molecular mechanism of this plasticity remains uncertain. In the CB, there are wide spread synaptic contacts not only between afferent nerve endings and type I cells, but also between neighboring type I cells [28]. Thus, the changes in the weight or strength of these synapses, which exist at multiple sites in the CB, are implicated in the plasticity of CB response to hypoxia. Studies have shown that CB contains a number of neurotransmitters and neuromodulators [29], such as dopamine, acetylcholine, ATP, nitric oxide, angiotensin, 5-HT. ATP and acetylcholine play an excitatory role by acting on the P2X2/3 receptors or nicotine acetylcholine receptors, whereas dopamine plays an inhibitory role by stimulating postsynaptic dopamine D2 receptor. Interestingly, glutamate, as a major excitatory neurotransmitter in the central nervous system, has not been significantly studied in the CB. As early as 1990, Torrealba [30] first found that there was a large amount of glutamate present in cat CB type I cells by immunohistochemistry staining, and they [31] speculated that glutamate in cat CB might be acting as a metabolite rather than a neurotransmitter.

Glutamate is the most abundant amino acid in the central nervous system, which is involved in a variety of physiological synaptic transduction, and induces the changes in synaptic morphology and function. The essential factors for glutamatergic neurotransmitters to play a role include glutamate, glutamate receptors and downstream signal molecules, VGluTs and EAATs. In our previous study [23, 24] we found that NMDA and AMPA receptors might be involved in the response of CB to CIH. Our RT-PCR results from this study, together with our previous report, revealed the presence of all VGluT1–3 and EAAT1–3 transcripts in human CB (Fig. 1) and rat CB. Among these glutamate transporters, the proteins for VGluT1, VGluT3, EAAT2 and EAAT3 were detected in human and rat CB (Fig. 2). Of note, we demonstrated an inability to detect protein for VGluT2 and EAAT1 in human and rat CB, even though both proteins were detected in brain of positive control; therefore, indicating that mRNA transcripts of VGluT2 and EAAT1 might not be translated to the proteins in human and rat CB.

In the nervous system, the basic condition for the release of glutamate as a neurotransmitter is that glutamate is transported into the transmitter vesicles through the VGluTs [25]. There are three subtypes of VGluTs. VGluT1 and VGluT2 are mainly distributed in glutamatergic neurons, while VGluT3 is not only distributed in glutamatergic neurons, but also in non-glutamic neurons, such as cholinergic neurons, GABA neurons and so on. Based on the results of western blotting in the present study, VGluT3 is the major type of vesicular glutamate transporter in the CB. Immunostaining showed that VGluT3 was mainly expressed in CB type I cells (Fig. 4A and B). Thus, we speculate that glutamate may be released from CB type I cells to synaptic space as a neurotransmitter to influence the electrical signal transmission of chemoreceptors. However, further study is needed to clarify whether glutamate acts as a neurotransmitter in the CB.

EAATs are located on presynaptic membrane, synaptic vesicle and glial cell membrane. They are important for the recycling of excitatory amino acids, the termination of excitatory signals and the protection of nerve cells from excitotoxic damage. In order to maintain the physiological effect, glutamate, which is released into the synaptic gap, needs to be reuptake through EAATs located in the nerve terminals or glial cells to avoid glutamate excitotoxicity. In the central nervous system, about 80% of glutamate is removed by reuptake into nerve endings and 20% into glial cells. There are five subtypes of EAATs family [25], in which EAAT1-3 are mainly distributed in the central nervous system, while EAAT4 is limited to the cerebellum [32]. EAAT5 is mainly distributed in the retina [33]. EAAT1 and EAAT2 are mainly expressed in the cell membrane of astrocytes, and remove the glutamate. While, EAAT3, EAAT4 and EAAT5 are mainly expressed in neurons, and some researchers even think that EAAT3 is neuron-specific. In the present study, we found that both human and rat CBs express mRNAs of all EAATs, as well as proteins of EAAT2 and EAAT3. EAAT2 was distributed in both type I and type II cells in the CB (Fig. 4C and D). However, EAAT3 was mainly expressed in type I cells (Fig. 4E and f), and EAAT2 seemed to have no cell heterogeneity. In any case, the expression of EAATs in CB may play an important role in synaptic physiological signal transmission between type I cells and nerve endings, or between type I cells.

**Fig. 6:**
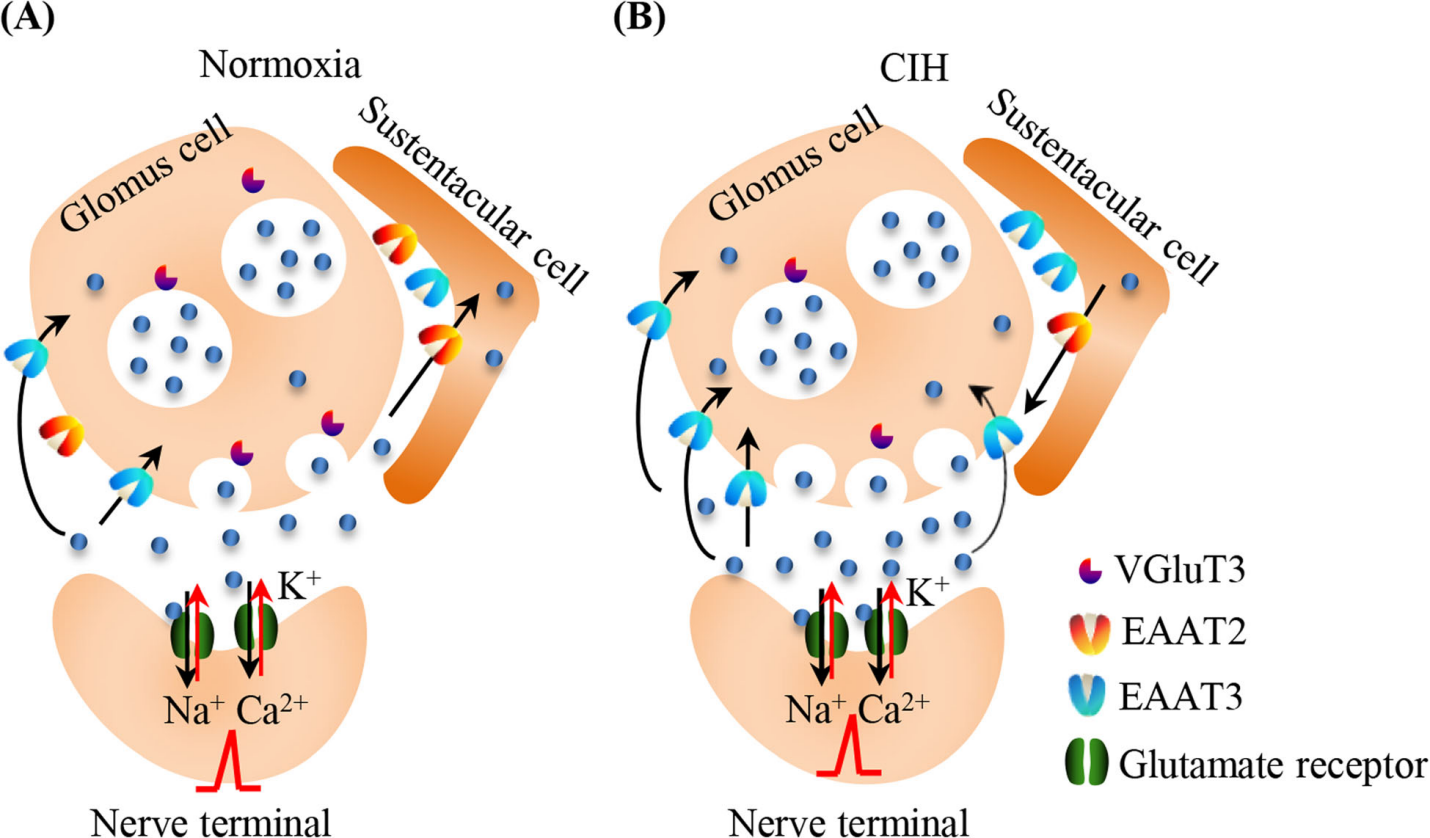
Schematic representation of glutamatergic neurotransmission in the CB. **a**. Under normoxia, glutamate released from CB pre-synaptic type I cell acts on glutamate ionotropic receptors on its corresponding post-synaptic type I cell. The influx of Na^+^ and Ca^2+^ ions cause depolarization and generation of action potential. **b**. CIH causes CB neuronal damages, then accumulation of glutamate induced neurotoxicity, leading compensatory reduction of VGluT2 and EAAT2 as well as an increase of EAAT3

It was found that hypoxia could promote glutamate secretion, increase extracellular glutamate concentration and glutamate excitatory toxicity [34]. In this study, we also found that CIH decreased the expression levels of VGluT3 and EAAT2, but increased the expression level of EAAT3 (Fig. 5). Accumulated evidences demonstrate that astrocytes can release glutamate [35–37], and glutamate reuptake induced by EAATs depends on the Na^+^ influx of Na^+^-K^+^ ATP enzyme. Under hypoxia, ATP synthesis was decreased, which leads to the reverse transport of EAATs, resulting in the release of glutamate from astrocytes to the synaptic space [36]. It was also found that GABA presynaptic neurons could reuptake glutamate into cells through EAAT3, providing glutamate for GABA synthesis [38]. Based on our findings, we speculate that hypoxia damages the integrity of the cell membrane after CIH treatment for 2 weeks, and a large amount of glutamate is released from type I cells into the synaptic space (Fig. 6). Similarly, hypoxia reverses the ability of EAAT2 to reuptake glutamate, resulting in the release of glutamate from type II glial cells. In order to prevent this toxicity, the expression levels of VGluT3 and EAAT2 are decreased as compensation. However, the expression of EAAT3 is increased compensatively, and subsequently causes reuptake of glutamate into type I cells. On the one hand, these changes protect type I cells from hypoxia injury by reducing release of glutamate and increasing reuptake of glutamate. On the other hand, reuptake of glutamate provides essential amino acids for synthesis of GABA, which as an important inhibitory neurotransmitter. GABA can reduce the excitatory neurotoxicity caused by excessive glutamate and protect cells from death.

Previous reporters demonstrated the differences of cellular responses to chronic sustained hypoxia (CSH) and CIH [39, 40], which might be due to the inhibition of mitochondrial function reversing the hypoxia-induced signal pathway [39, 41]. Under chronic continuous hypoxia, mitochondria consume almost all the oxygen, resulting stabilization of HIF-1α (hypoxia-inducible factor-1α) and transcription of target genes. While during intermittent hypoxia, due to mitochondrial stress, superoxide anions are not to stabilize HIF-1α, but activate the inflammatory pathway. Peng et al. [14] also found that CIH (5% O_2_ 68–75 s and 21% O_2_ 70–85 s for 8 h/day, 10 days) rather than CSH (5% O_2_ for 4 h/day, 10 days) elicited CB chemoreceptor plasticity. Thus, the regulatory effects and underlying mechanisms of CIH and CSH on VGluTs and EAATs in the rat CB may be different. Further works remain to be completed to further specify these differences.

Although this study defined the expression of VGluTs and EAATs in the CB and the influence of CIH on the levels of these proteins, two major limitations deserve comment. First, we did not characterize the functional significances and associated mechanisms of VGluTs and EAATs in CB response or carotid chemoreflex plasticity following CIH exposure. There is definite evidence that VGluTs or EAATs are involved in glutamate-mediated synaptic plasticity [27, 42–44]. Future studies involving carotid sinus nerve electrophysiological recording along with the manipulating the level of glutamate transporters in the CB will hopefully clarify the actions of glutamate transporters on chemoreceptor response to CIH, CSH as well as acute hypoxia. A second limitation of current study is that we did not examine the level of glutamate transporter proteins in the CB from OSA patients. Although CIH rat model has been widely used to study the role of CB in OSA-induced hypertension [4–6], and current study also shows the similar expression features of glutamate transporters in rat and human CB, there is considerable evidence that some characteristics of glomus cells are species specific [45–47]. Thus, this study could not conclusively ensure that the response of human CB to CIH is qualitatively the same as that observed for the rat CB. Future work involving the study of human CB specimens from OSA patients will clinically elucidate the pathophysiological aspect of glutamate transporter proteins in human CB exposed to CIH.

## Conclusions

In summary, our study found that the multiple VGluTs and EAATs are expressed in human and rat CBs, which indirectly demonstrates that glutamate exists as a neurotransmitter in the CB. Our data also indicate that VGluT3, EAAT2 and EAAT3 are functionally expressed in the CB, and may be involved in the response of the CB to hypoxia.

## Data Availability

All data generated or analyzed during this study are included in this article.

## Abbreviations

AMPA1: Alpha-amino-3-hydroxy-5-methyl-4-isoxazole propionic acid receptor 1
CB: Carotid body
CIH: Cyclic intermittent hypoxia
CSH: Chronic sustained hypoxia
EAAT: Excitatory amino acid transporter
GFAP: Glial fibrillary acidic protein
HIF-1α: Hypoxia-inducible factor-1α
NMDAR1: N-methyl-D-aspartate Receptor 1
OSA: Obstructive sleep apnea
VGluT: Vesicular glutamate transporter
TH: Tyrosine hydroxylase
RT: Reverse transcription

## References

[1] Nieto FJ, Young TB, Lind BK, Shahar E, Samet JM, Redline S, D'Agostino RB, Newman AB, Lebowitz MD, Pickering TG (2000). Association of sleep-disordered breathing, sleep apnea, and hypertension in a large community-based study. Sleep Heart Health Study JAMA.

[2] Sjöström C, Lindberg E, Elmasry A, Hägg A, Svärdsudd K, Janson C (2002). Prevalence of sleep apnoea and snoring in hypertensive men: a population based study. Thorax..

[3] Drager LF, Genta PR, Pedrosa RP, Nerbass FB, Gonzaga CC, Krieger EM, Lorenzi-Filho G (2010). Characteristics and predictors of obstructive sleep apnea in patients with systemic hypertension. Am J Cardiol.

[4] Fletcher EC, Lesske J, Qian W, Miller CC, Unger T (1992). Repetitive, episodic hypoxia causes diurnal elevation of systemic blood pressure in rats. Hypertension..

[5] Fletcher EC, Lesske J, Culman J, Miller CC, Unger T (1992). Sympathetic denervation blocks blood pressure elevation in episodic hypoxia. Hypertension..

[6] Fletcher EC, Lesske J, Behm R, Miller CC, Stauss H, Unger T (1992). Carotid chemoreceptors, systemic blood pressure, and chronic episodic hypoxia mimicking sleep apnea. J Appl Physiol (1985).

[7] Shirahata M, Balbir A, Otsubo T, Fitzgerald RS (2007). Role of acetylcholine in neurotransmission of the carotid body. Respir Physiol Neurobiol.

[8] Iturriaga R (2014). Functional studies of acetylcholine, ATP and cytokine release from the human carotid body: the new frontier for oxygen chemoreception physiology. Exp Physiol.

[9] Welsh MJ, Heistad DD, Abboud FM (1978). Depression of ventilation by dopamine in man. Evidence for an effect on the chemoreceptor reflex. J Clin Invest.

[10] Ureña J, Fernández-Chacón R, Benot AR, Alvarez de Toledo GA, López-Barneo J (1994). Hypoxia induces voltage-dependent Ca2+ entry and quantal dopamine secretion in carotid body glomus cells. Proc Natl Acad Sci U S A.

[11] Platero-Luengo A, González-Granero S, Durán R, Díaz-Castro B, Piruat JI, García-Verdugo JM, Pardal R, López-Barneo J (2014). An O_2_-sensitive glomus cell-stem cell synapse induces carotid body growth in chronic hypoxia. Cell..

[12] Murali S, Zhang M, Nurse CA (2014). Angiotensin II mobilizes intracellular calcium and activates pannexin-1 channels in rat carotid body type II cells via AT1 receptors. J Physiol.

[13] Fung ML (2016). Angiotensin II in the carotid body - a friend or foe?. Exp Physiol.

[14] Peng YJ, Overholt JL, Kline D, Kumar GK, Prabhakar NR (2003). Induction of sensory long-term facilitation in the carotid body by intermittent hypoxia: implications for recurrent apneas. Proc Natl Acad Sci U S A.

[15] Feldman JL, Mitchell GS, Nattie EE (2003). Breathing: rhythmicity, plasticity, chemosensitivity. Annu Rev Neurosci.

[16] Prabhakar NR (2011). Sensory plasticity of the carotid body: role of reactive oxygen species and physiological significance. Respir Physiol Neurobiol.

[17] Gonzalez C, Vaquero LM, López-López JR, Pérez-García MT (2009). Oxygen-sensitive potassium channels in chemoreceptor cell physiology: making a virtue of necessity. Ann N Y Acad Sci.

[18] Ortiz F, Iturriaga R, Varas R. Sustained hypoxia enhances TASK-like current inhibition by acute hypoxia in rat carotid body. Adv Exp Med Biol. 2009;648:83–8. 10.1007/978-90-481-2259-2_9 19536468

[19] Di Giulio C, Verratti V, Artese L, Petruccelli G, Walski M, Pokorski M (2009). Aging and expression of heme oxygenase-1 and endothelin-1 in the rat carotid body after chronic hypoxia. J Physiol Pharmacol.

[20] Moreau JM, Messenger SA, Ciriello J (2015). Effects of angiotensin II on leptin and downstream leptin signaling in the carotid body during acute intermittent hypoxia. Neuroscience..

[21] Kato K, Yokoyama T, Yamaguchi-Yamada M, Yamamoto Y (2013). Short-term hypoxia transiently increases dopamine β-hydroxylase immunoreactivity in glomus cells of the rat carotid body. J Histochem Cytochem.

[22] Bialkowska M, Zajac D, Mazzatenta A, Di Giulio C, Pokorski M (2015). Inhibition of peripheral dopamine metabolism and the ventilatory response to hypoxia in the rat. Adv Exp Med Biol.

[23] Liu Y, Ji ES, Xiang S, Tamisier R, Tong J, Huang J, Weiss JW (2009). Exposure to cyclic intermittent hypoxia increases expression of functional NMDA receptors in the rat carotid body. J Appl Physiol (1985).

[24] Liu Y, Li C, Jia X, Huang L, Weiss JW (2018). AMPA receptor-dependent Glutamatergic signaling is present in the carotid chemoreceptor. Neuroscience..

[25] Shigeri Y, Seal RP, Shimamoto K (2004). Molecular pharmacology of glutamate transporters, EAATs and VGLUTs. Brain Res Brain Res Rev.

[26] Danbolt NC (2001). Glutamate uptake. Prog Neurobiol.

[27] Watanabe M (2013). Glutamate signaling and neural plasticity. No To Hattatsu.

[28] Morgan M, Pack RJ, Howe A (1975). Nerve endings in rat carotid body. Cell Tissue Res.

[29] Nurse CA (2014). Synaptic and paracrine mechanisms at carotid body arterial chemoreceptors. J Physiol.

[30] Torrealba F (1990). Immunocytochemistry of neuroactive substances in visceral receptors: glutamate and CGRP. Arch Biol Med Exp.

[31] Torrealba F, Bustos G, Montero VM (1996). Glutamate in the glomus cells of the cat carotid body: immunocytochemistry and in vitro release. Neurochem Int.

[32] Bridges RJ, Esslinger CS (2005). The excitatory amino acid transporters: pharmacological insights on substrate and inhibitor specificity of the EAAT subtypes. Pharmacol Ther.

[33] Pow DV, Barnett NL (2000). Developmental expression of excitatory amino acid transporter 5: a photoreceptor and bipolar cell glutamate transporter in rat retina. Neurosci Lett.

[34] Choi DW, Rothman SM (1990). The role of glutamate neurotoxicity in hypoxic-ischemic neuronal death. Annu Rev Neurosci.

[35] Szatkowski M, Barbour B, Attwell D (1990). Non-vesicular release of glutamate from glial cells by reversed electrogenic glutamate uptake. Nature..

[36] Rossi DJ, Oshima T, Attwell D (2000). Glutamate release in severe brain ischaemia is mainly by reversed uptake. Nature..

[37] Back SA, Rosenberg PA (2014). Pathophysiology of glia in perinatal white matter injury. Glia..

[38] Stafford MM, Brown MN, Mishra P, Stanwood GD, Mathews GC (2010). Glutamate spillover augments GABA synthesis and release from axodendritic synapses in rat hippocampus. Hippocampus..

[39] Yeo EJ (2019). Hypoxia and aging. Exp Mol Med.

[40] Brown ST, Buttigieg J, Nurse CA (2010). Divergent roles of reactive oxygen species in the responses of perinatal adrenal chromaffin cells to hypoxic challenges. Respir Physiol Neurobiol.

[41] Taylor CT (2008). Mitochondria and cellular oxygen sensing in the HIF pathway. Biochem J.

[42] Divito CB, Underhill SM (2014). Excitatory amino acid transporters: roles in glutamatergic neurotransmission. Neurochem Int.

[43] Valtcheva S, Venance L (2019). Control of long-term plasticity by glutamate transporters. Front Synaptic Neurosci.

[44] Gonçalves-Ribeiro J, Pina CC, Sebastião AM, Vaz SH (2019). Glutamate transporters in hippocampal LTD/LTP: not just prevention of Excitotoxicity. Front Cell Neurosci.

[45] Barer G (1994). Carotid bodies in animal models of human disease: what do they teach us?. Thorax.

[46] Dvorakova MC, Kummer W (2005). Immunohistochemical evidence for species-specific coexistence of catecholamines, serotonin, acetylcholine and nitric oxide in glomus cells of rat and Guinea pig aortic bodies. Ann Anat.

[47] Fagerlund MJ, Kåhlin J, Ebberyd A, Schulte G, Mkrtchian S, Eriksson LI (2010). The human carotid body: expression of oxygen sensing and signaling genes of relevance for anesthesia. Anesthesiology..

